# Rapid construct superhydrophobic microcracks on the open-surface platform for droplet manipulations

**DOI:** 10.1038/s41598-021-94484-y

**Published:** 2021-07-21

**Authors:** Wan-Hsuan Lin, Chien-Wei Chen, Sheng-Hang Wang, Bor-Ran Li

**Affiliations:** 1 Institute of Biomedical Engineering, College of Electrical and Computer Engineering, National Yang Ming Chiao Tung University, 1001 Ta-Hseh Rd., Hsinchu, Taiwan; 2 Department of Electrical and Computer Engineering, College of Electrical and Computer Engineering, National Yang Ming Chiao Tung University, Hsinchu, Taiwan; 3 Taiwan Instrument Research Institute, National Applied Research Laboratories, Hsinchu, Taiwan; 4 Center for Emergent Functional Matter Science, National Yang Ming Chiao Tung University, Hsinchu, Taiwan

**Keywords:** Chemistry, Engineering

## Abstract

Droplet-based transport driven by surface tension has been explored as an automated pumping source for several biomedical applications. This paper presented a simple and fast superhydrophobic modify and patterning approach to fabricate various open-surface platforms to manipulate droplets to achieve transport, mixing, concentration, and rebounding control. Several commercial reagents were tested in our approach, and the Glaco reagent was selected to create a superhydrophobic layer; laser cutters are utilized to scan on these superhydrophobic surface to create gradient hydrophilic micro-patterns. Implementing back-and-forth vibrations on the predetermined parallel patterns, droplets can be transported and mixed successfully. Colorimetry of horseradish peroxidase (HRP) mixing with substrates also reduced the reaction time by more than 5-times with the help of superhydrophobic patterned chips. Besides, patterned superhydrophobic chips can significantly improve the sensitivity of colorimetric glucose-sensing by more than 10 times. Moreover, all bioassays were distributed homogeneously within the region of hydrophilic micropatterns without the coffee-ring effect. In addition, to discuss further applications of the surface wettability, the way of controlling the droplet impacting and rebounding phenomenon was also demonstrated. This work reports a rapid approach to modify and patterning superhydrophobic films to perform droplet-based manipulations with a lower technical barrier, higher efficiency, and easier operation. It holds the potential to broaden the applications of open microfluidics in the future.

## Introduction

Generally speaking, microfluidics devices are composed of closed channels, and lots of components (e.g., micropumps, microvalves, and other micromixers) are used to assist in completing complex liquid handling tasks^1–5^. By comparison, open-surface microfluidics mainly controls dispersed droplets on the open-surface device by adjusting surface structures and wettability^6–8^. In addition, open surface microfluidics offers several advantages over the conventional counterpart, including simple monolithic construction, direct environmental accessibility, no cavitation/interfacial obstruction, clear optical path, and compatibility with biological experiments^9^. In recent years, open-surface microfluidics has attracted much attention due to several advantages, such as reduced consumption of reagents and samples^10^; a simplified integration processes and controlled system (i.e., no bonding is required); and most importantly, no channels exist, thereby avoiding trapped bubbles and eliminating the risk of the microchannel clogging^11–13^. Thus, open-surface microfluidics has great potential in point-of-care (POC) diagnostics and lab-on-a-chip (LOC) applications^14,15^.

Droplet-based microfluidic manipulations have recently been applied to a variety of biomedical analyses and point-of-care diagnostics^16–18^. Minute reaction volume of the droplet operations on microfluidic platforms enable novel implementations of high-throughput screening, massive biochemical synthesis, and high-efficiency parallel reactions^19^. However, a driving droplet with micropumps built on the conventional microfluidic platform is not easy work. So far, many actuation mechanisms have been utilized to control the droplet movement on an open surface, including electrowetting-on-dielectric (EWOD)^20^, acoustic wave^21,22^, or actuation by mechanical agitation^23^. These approaches might control droplets well, but the system is still complicated with delicate design and operations^24^. Therefore, over the past few years, several artless approaches have been proposed to manipulate droplets.

One of the latest developments is patterned superhydrophobic open-surface microfluidics, which offers an alternative means to manipulate droplets with the “Lotus effect”^9,25^. Meanwhile, the behavior of droplets on superhydrophobic surfaces^26–28^ and related analytical methods^29,30^ have been studied. Various surface modification techniques are used to fabricate superhydrophobic surface, including UV irradiation, plasma polymerization, polymer coating, electrospinning, chemical vapor deposition, and photolithography^31,32^. However, most methods require multistep fabrication or surface treatments, and some of the superhydrophobic surfaces are not transparent, limiting the applications for extensive research. Furthermore, these technologies might be inconvenient to use. In the past decade, the wettability of surface modification with superhydrophobicity have been utilized in scientific research and practical applications, such as self-cleaning, anti-icing, anti-corrosion, fuel transfer, nuclear textiles, and many more. Based on the fact that surface roughness and low surface energy materials are important factors affecting surface wettability, patterning would be a simple way to help increase the surface wettabiity by exposing some portions of the glass surface to the droplets.

This study presented a rapid approach to modify and pattern a superhydrophobic film on an open-surface substrate with advantages such as low cost, transparency, high uniformity, and simple fabrication on a large scale. By depositing the superhydrophobic material on a glass substrate and using laser irradiation to create wettability patterns, this platform successfully demonstrated various droplet operations, including transport of multiple droplets, mixing of two liquid states droplets, the concentration of multiple droplets, and exploring the rebounding phenomenon of a droplet on a wettable superhydrophobic surface. As a proof of concept, we present the colorimetric assay to show the rapid mixing of HRP/TMB solution and exhibit how to detect the concentration of glucose on our platform.

## Results and discussion

### Characterizations of the superhydrophobic film

**Figure 1:**
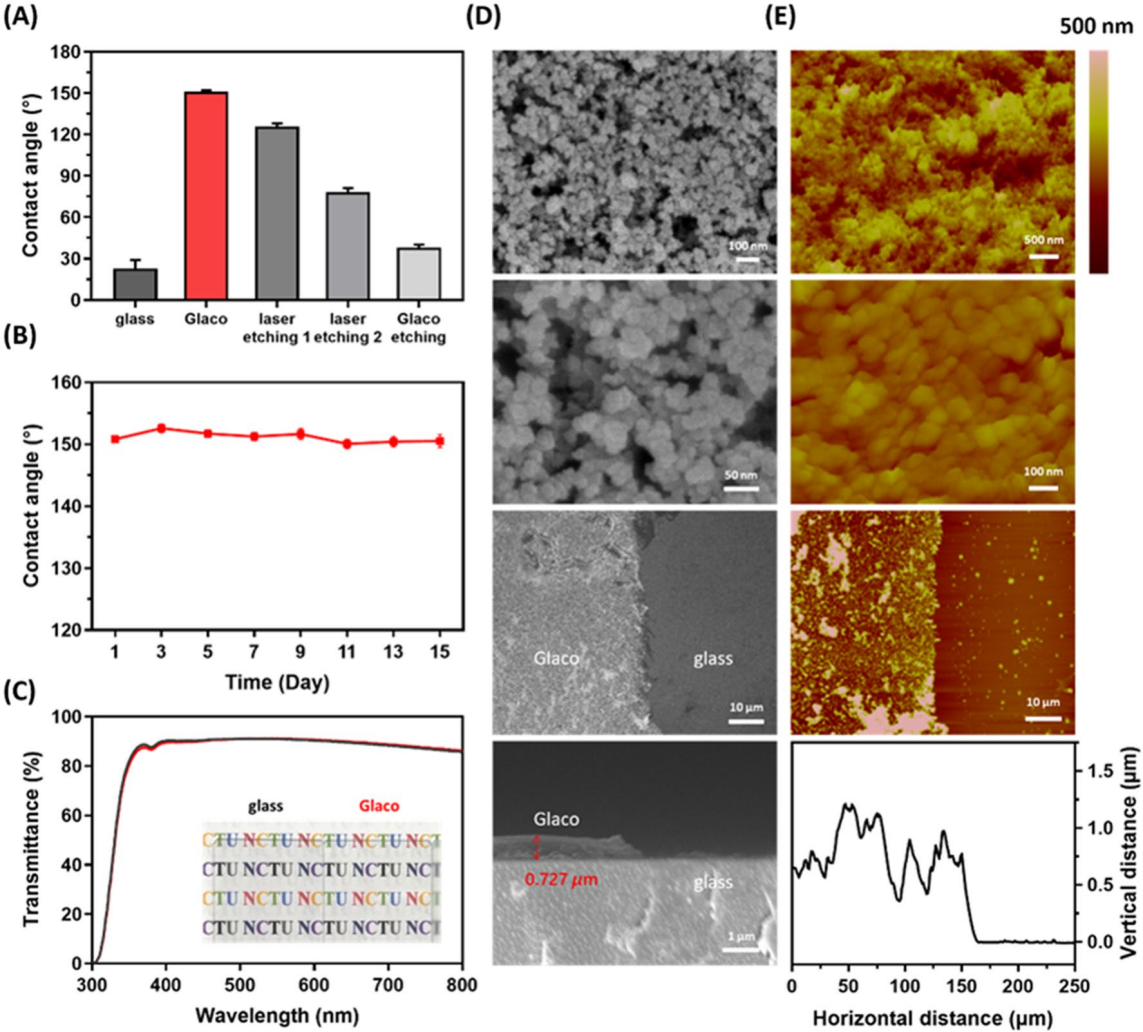
Characterizations of the superhydrophobic film. (**A**) The contact angle of the bare glass, Glaco-modified coating on the glass and the glass with laser patterns. (**B**) Stability of the superhydrophobic film. (**C**) Investigation of the transmittance of glass and Glaco-modified film. (**D**) SEM images (top-view, side-view) of Glaco-modified surface, including different zooms and thickness of ∼0.7 µm. (**E**) Corresponding profile of AFM scan showing the size and structure of the Glaco nanoparticles and clusters.

Without Glaco-modified coating, the water droplet placed on a glass slide exhibited an intrinsic contact angle of approximately 22°. After the superhydrophobic film was coated on the glass substrate, and the contact angle increased to 150°. Then, we were able to control the droplets’ contact angle between 22° and 150° by adding the designed laser patterns upon the Glaco film (Fig. 1A). For most biometric applications, the superhydrophobic film should be stable enough as an operating platform. It was found that our superhydrophobic film has good surface stability to maintain the water contact angle at 151 ± 0.8° over 2 weeks (Fig. 1B). To study the potential of optical detection, the optical transmittance of the superhydrophobic film had been analyzed in the visible spectrum. The adsorption of the surface was investigated by UV–visible spectrometry in the wavelength range of 400–700 nm (Fig. 1C). The transmittance of the Glaco coated glass substrate was ~ 90%, which was almost the same as the pure glass slide. In other words, the Glaco-modified surface had high transparency, which does not affect visual observation with the naked eye.

After the superhydrophobic film was coated with a commercial agent (Glaco Mirror Coat “Zero”), an alcohol-based suspension of silica nanoparticles, the morphologies of the surface were investigated by Scanning Electron Microscopy (SEM) and Atomic Force Microscopy (AFM). Figure 1D provides the SEM images of the superhydrophobic substrate, consisting of nanoparticles coating with a thickness of ∼0.7 µm. Figure 1E displays the topology of the superhydrophobic surface as observed by AFM. The particle size was about 30–50 nm, and the roughness (Rq) of the superhydrophobic film was 50–200 nm. SEM images of the arrangement of points created by laser cutter could be found in the Figure S1 in the supporting information.

### Transport of a single droplet

**Figure 2:**
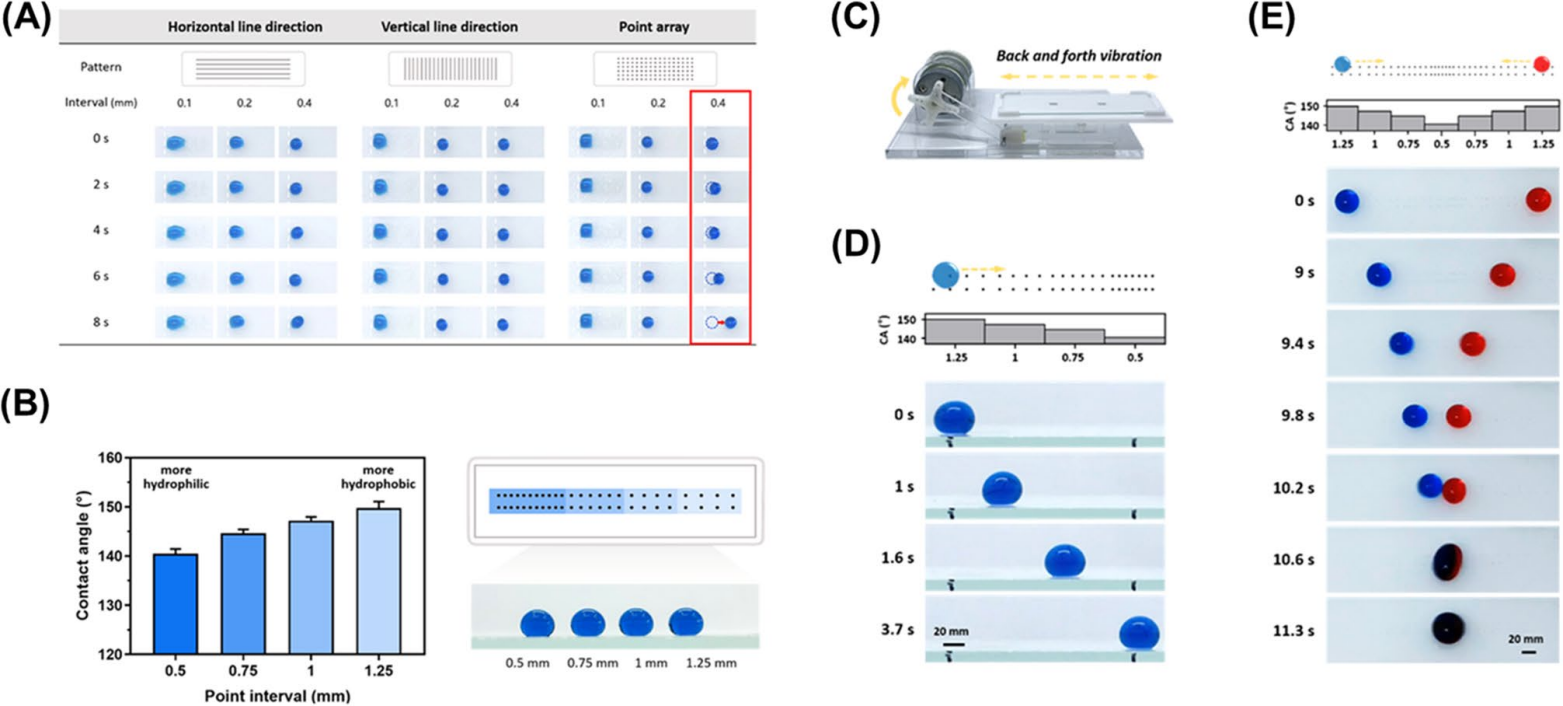
(**A**) Contact angle measurements with a horizontal line, vertical line, and point array. (**B**) Contact angle measurements with different intervals of point cascade (0.5, 0.75, 1, 1.25 mm). The red square indicates the optimized conditions. (**C**) The working mechanism of the droplet actuation system. (**D**) Time-lapsed images (side-view) of a 10 μL droplet transport. (**E**) Time-lapsed images (top-view) of droplets mixing with two 10 μL droplets on a mixing point cascade from opposite sides.

We set several kinds of point and line intervals range in a cascade to adjust the surface wettability to control the droplet movements (Fig. 2A). The water contact angles are 140.5 ± 0.9° with 0.5 mm point interval, 144.6 ± 0.7° with 0.75 mm point interval, 147.2 ± 0.7° with 1 mm point interval, and 149.8 ± 1.3° with 1.25 mm point interval. (Fig. 2B) The static contact angle of the superhydrophobic surface without laser irradiation is 151 ± 0.6°. The schematic of the transport mechanism is provided in Fig. 2C. When the actuation system is applied, it would generate a back-and-forth mechanical force to overcome the inertial force and drag force between droplet and surface, then drive the droplet to the predestinate position. Under the small vibration frequency, *F*_*inert*_ cannot overcome the *F*_*d*_ so that the droplet remains stationary. Once the vibration frequency exceeds a certain threshold, *F*_*inert*_ overcomes the *F*_*d*_ then the droplet starts to move to generate the acceleration against travel^33^. The droplet starts to accelerate, as described by:

$$ F_{inert} - F_{d} = m\frac{{d^{2} x}}{{dt^{2} }}. $$

Following the previous research, the inertial force can be expressed as $F_{inert} = mA_{0} \left( {2\pi f} \right)^{2} \sin 2\pi ft$, where *m* is the mass of the droplet, *A*_*0*_ is the vibration amplitude constant in this study, and *f* is the vibration frequency. *F*_*inert*_ is affected by the vibration frequency *f,* and they are in direct proportion. The drag force meaning the hysteresis resistance of the droplet on the hydrophilic micropattern refers to $F_{d} = \gamma Cw(\cos \theta r - {\text{cos}}\theta a)$, where *γ* is the surface tension of water/air interface, *C* is a shape-correction coefficient related to the geometry of the contact line between the droplet and the substrate, *w* is the pattern width of point cascades, and *θ*r and *θ*a refer to the dynamic contact angle of advancing edge and receding edge of the droplet respectively. We found that to move the droplets along specific directions; we need to control the wettability of hydrophilic micropatterns on the superhydrophobic surface. It confirms that the variable point interval created by laser can generate gradient surface wettability. The transport of droplets was demonstrated with a 10 μL droplet (Fig. 2D). If at the beginning of the back-and-forth vibration was applied, the droplet would stay at the more hydrophobic edge of the pattern. As the vibration frequency exceeded 3 Hz, the droplet would overcome the drag force of the 1.25 mm point interval pattern and start to move forward. The droplet would move directly to the more hydrophilic edge step by step from the more hydrophobic edge until it could not overcome the drag force of the 0.5 mm point interval pattern. The droplet can travel stably over a distance of 18.5 mm within 3.7 s.

### Droplet mixing

The ability to mix two droplets uniformly and quickly is one of the most important functions in open channel microfluidics for biochemical assays^26–29^. The droplet mixing pattern consists of two-point cascades; the point intervals are 0.5 (more hydrophilic edge), 0.75, 1, and 1.25 mm (more hydrophobic edge). When the back-and-forth vibrations were applied, droplets would gradually move to the middle from the opposite sides of the pattern. Figure 2E shows an image of mixing performed on two 10 μL droplets. Water droplets stained with different colors (blue and red) were dispensed at the ends of the two opposite cascades. Under the vibration of 3 Hz, these droplets on opposite sides of the pattern moved to the middle towards the most hydrophilic edge to merge because of the gradient surface wettability. Finally, the merged droplet stocked stably in the middle of the pattern, and the color gradually changed. The process of moving and mixing the droplets took approximately 11.3 s.

**Figure 3:**
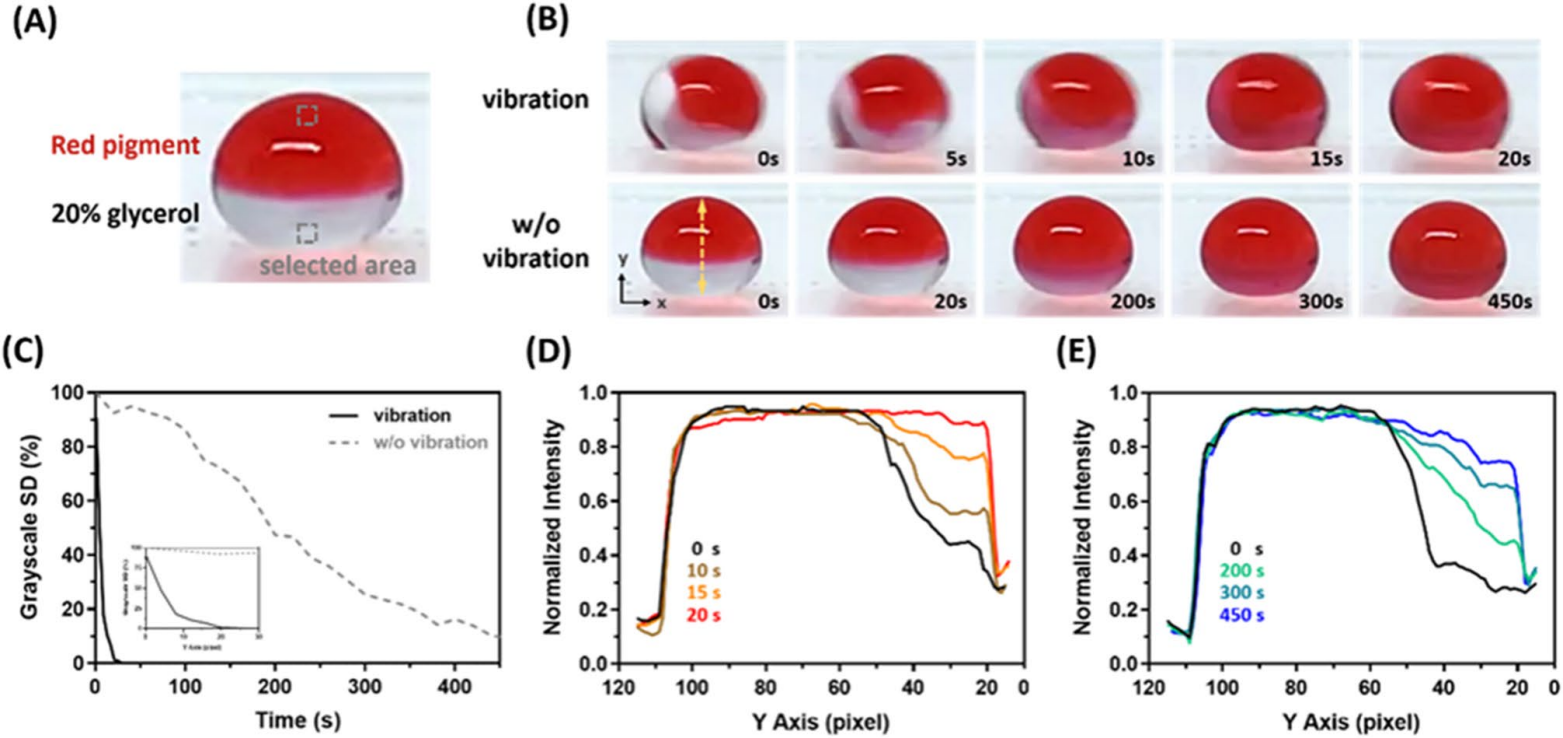
(**A**) The droplet mixture uses a red pigment and 20% glycerol to demonstrate. (**B**) Time-lapsed images of the droplet mixing process. (**C**) Calculating the selected area for the standard deviation of the grayscale image indicates the droplet mixing efficiency, and the distributions of normalized intensity along the yellow dashed line (**B**) indicate the color of the mixture droplet changing over time with (**D**) and without (**E**) vibration.

In many biological experiments, lots of biological samples are stored in glycerol. Thus, rapid and homogeneous mixing of two liquid solutions becomes more important^30^. After confirming the mixing capability of two water droplets, the efficiency of droplet mixing is investigated using red pigment and glycerol mixtures that dissolve in DI water. Figure 3A shows the droplets mixing under the vibrational frequency of 3 Hz, 10 μL red pigment located on the upper side of the droplet and 10 μL 20% glycerol on the lower side. The vibration generated an internal flow to promote the mixing of two chemicals inside the droplet. The two chemicals mixed completely within 20 s. However, the droplet took over 7 min to mix homogeneously, relying solely on passive diffusion, as shown in Fig. 3B. The mixing efficiency was evaluated through ImageJ analysis. The color image was converted to grayscale, which is then used to calculate the value of the selected area and the standard deviation. The standard deviation of the grayscale image decreased with more uniform droplet mixing. The droplet mixing data were collected and presented in Fig. 3C. The droplet mixing efficiency with vibration is 20 times higher than without vibration. The distributions of normalized intensity along the yellow dashed line in Fig. 3B indicate the mixture droplet’s color gradually changed over time. The mixture droplet exhibits a homogeneous intensity distribution after 20 s with vibration (Fig. 3D). On the other hand, Fig. 3E shows the normalized intensity of the mixture droplet distributed homogeneously over 450 s without the vibration.

Similar mixing efficiency also demonstrated by TMB and HRP (Fig. S2). The HRP concentration was 0.01 mg/mL. The mixture droplet consisted of 10 μL TMB solution located on the upper side of the droplet and 10 μL HRP in 20% glycerol on the lower side (Fig. S2A). After the two chemicals were mixed under the vibrational frequency of 3 Hz, HRP started to react with TMB solution and showed a blue color within 20 s, and mixed homogeneously in 80 s. However, the droplet could not mix evenly when depending on passive diffusion without vibration. When HRP excessively reacted with TMB solution, it would appear yellow at the boundary of two chemicals (Fig. S2B). The mixing efficiency was evaluated through ImageJ analysis, and the color image was converted to grayscale. Then, the grayscale value of the selected areas was calculated. If the droplet becomes homogeneous, the lower side of the mixture droplet will turn blue. The mixing efficiencies of the mixture droplets (grayscale change) were measured and plotted in Figure S2C. The distribution of colors on a pixel was made up of three signals: red, green, and blue (RGB). By describing their corresponding intensities, the reaction of HRP with TMB was indicated along the yellow dashed line in Figure S2B. Before 80 s, the mixture droplet with vibration presented a homogeneous blue intensity (Fig. S2D); the mixture droplet without vibration exhibited a color shift from blue to green to yellow (Fig. S2E). The vibration not only brought the two droplets together but also enhanced the droplet mixing efficiency. The color of the mixed droplet became homogeneous with vibration within 80 s, which is more than 5 times faster than mixing without vibrations.

### Droplet concentrates on wettability micropattern

To demonstrate the vast applicability of the superhydrophobic platform for extensive bio-applications, we improved the reliability of biochips, focusing on spot homogeneity. The common phenomenon of coffee-ring morphology has often been observed on the substrate, which may cause inhomogeneous signals and even lead to inaccurate and unreliable readouts. Therefore, it is vital to control the spot homogeneity at the sensing interface.

**Figure 4:**
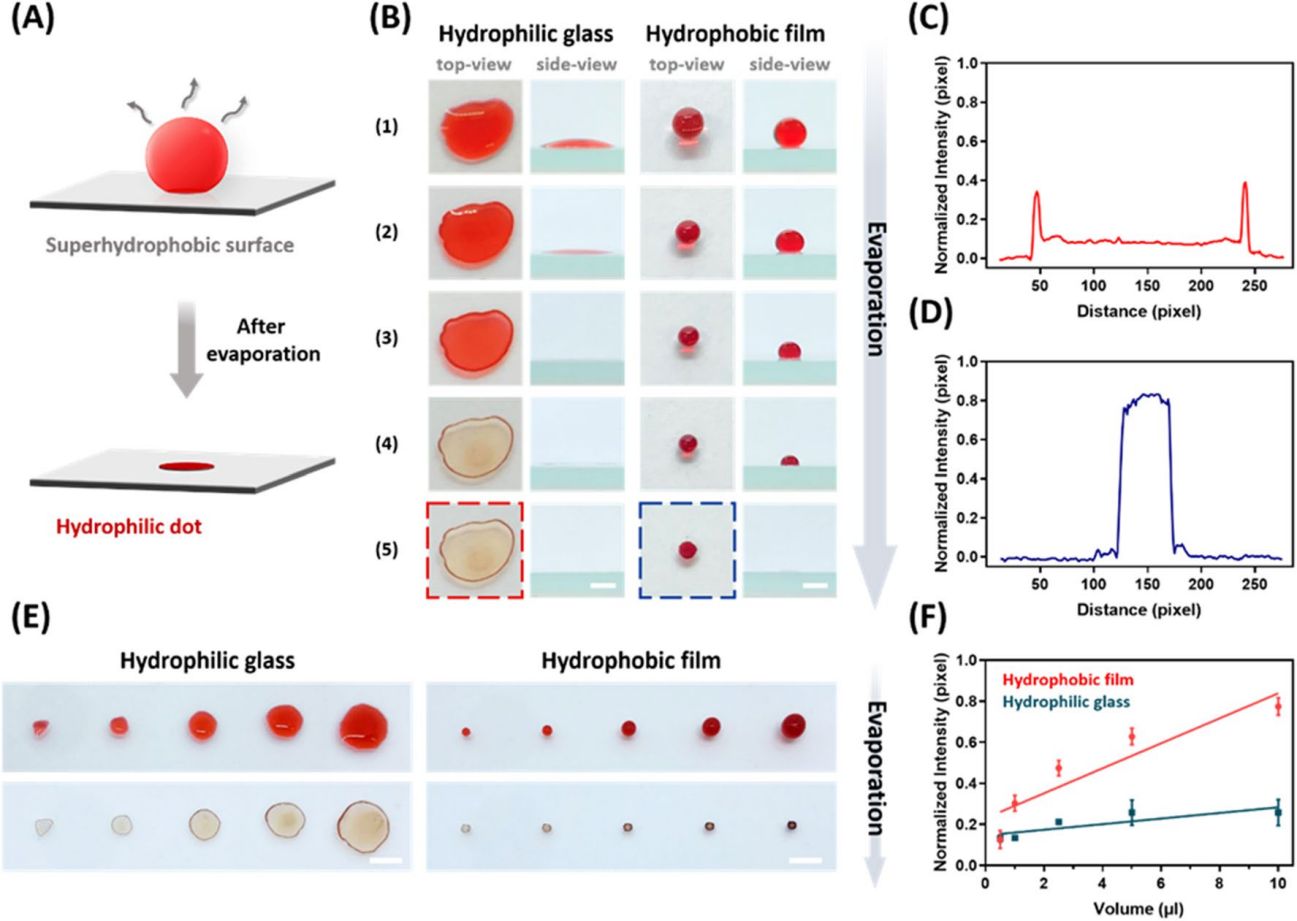
(**A**) Schematic illustration of the droplet returning to a homogeneous deposition after droplet evaporation. (**B**) Morphology change (top-view and side-view) of 10 μL droplet evaporation on the hydrophilic glass and hydrophobic film (the scale bar is 10 mm). (**C**) The intensity analysis of deposit dot on hydrophilic glass. (**D**) The intensity analysis of deposited dot on hydrophobic film. (**E**) The optical images of droplet evaporation deposited on different substrates with different droplet volumes (the scale bar is 20 mm). (**F**) The relationship between deposited dot intensity and droplet volume is based on the different substrates.

A 1-mm diameter hydrophilic pattern was created on a superhydrophobic surface by laser irradiation. A Schematic illustration is shown in Fig. 4A. The 10 μL droplets on this hydrophilic pattern would become homogeneous through evaporation. During the evaporation process, the coffee-ring effect always occurred on hydrophilic glass. The irregular shape of the droplet pinned on the surface and the base diameter remained roughly the same (diameter of 6.29 ± 0.7 mm). However, the droplet on the hydrophobic film was trapped in the hydrophilic dot because the hydrophobic film formed a circle barrier that limited the liquid expansion and the droplet concentrated homogeneously in a 1 mm diameter pattern (Fig. 4B). After the red pigment droplet evaporated on the hydrophilic glass, the droplet tended to deposit along the periphery of itself or spread over a larger area. The weak intensity led to difficulty detecting sensitive signal readouts (Fig. 4C). The intensity of the deposited pattern on the hydrophobic film improved the homogeneity of sample distribution and increased the sensitivity (Fig. 4D). The capability of the concentrating platform had been proven by comparing the results between Fig. 4D.

The laser-treated surface can carry different droplet sizes, including 0.5, 1, 2.5, 5, and 10 μL. The optical images of a deposited droplet on hydrophilic and hydrophobic substrates are shown in Fig. 4E. The detected intensity of the droplet image increased with an increasing volume of deposited droplets. Droplets on the hydrophobic surface have stronger detected intensities and lighter coffee-ring deposition after evaporation. Figure 4F shows the comparison of concentrated droplets detecting sensitivity between hydrophilic and hydrophobic platforms. The error bars for the intensity of deposited droplets on the hydrophilic glass were greater than that of the hydrophobic film.

### Detection of glucose colorimetric assay

**Figure 5:**
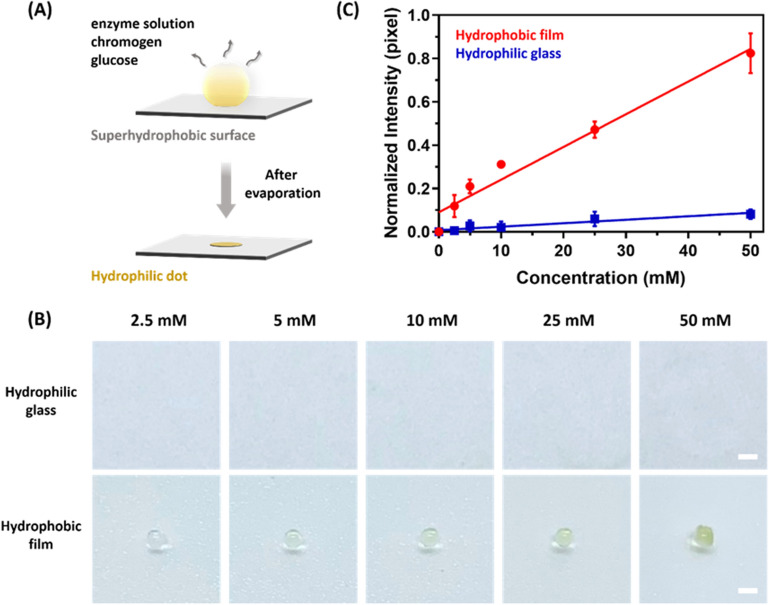
(**A**) Schematic diagram of droplet evaporation in glucose colorimetric assay. (**B**) Morphology change of mixture droplet evaporation on the hydrophilic glass and hydrophobic film with different concentrations (the scale bar is 1 mm). (**C**) The intensity analysis of deposited dot on different substrates.

The droplets with the enzyme solution were placed on the superhydrophobic film and evaporated to demonstrate the glucose colorimetric detection for surface applications. A schematic illustration of droplets’ colorimetric evaporation is shown in Fig. 5A. Colorimetric assays were performed on the droplets under different conditions. First, we placed 2 μL of enzyme solution droplets on the different substrates with chromogen, and then added 1 μL of glucose sample with concentration ranging from 2.5 to 50 mM. Compared to the mixture droplets on the hydrophilic glass, the mixture droplets on the superhydrophobic film with the hydrophilic dots evaporated at preset positions. The edge of droplets pinned on the hydrophilic dots had a 1-mm diameter, while the actual concentrated diameter was 1 ± 0.05 mm. The mixture droplets on the hydrophilic glass left a coffee ring due to liquid surface diffusion (diameter of 4.6 ± 0.5 mm). We conjected the reaction solution evaporated too quickly to react completely (Fig. 5B). After analyzing the intensity of hydrophilic dots after droplets concentrated on the different substrates, we found that the mixture droplets showed a significant increase in intensity when controlling the droplets on a hydrophobic film (Fig. 5C).

### Droplet rotational rebounding on a superhydrophobic surface

When a droplet meets a solid surface, it produces different results according to different surface properties like deposition or rebound. The wettability of the surface will change the rebounding direction of the droplet. There is great potential for driving liquid and directional transportation without external force control. The experimental system contains a syringe pump (NE-300, GrandHand, Taiwan) to produce the droplets and a high-speed camera (PROMON U1000, AOS Technologies AG, Switzerland) to record the impacting process of the droplets. Wettability patterns on the superhydrophobic surface were created by laser irradiation, as described previously. This surface is located horizontally on an XY freedom platform. Generally, a droplet falls due to gravity until it impacts the superhydrophobic surface without any wettability patterns. It typically spreads into a liquid film and then rebounds vertically upward (Fig. S3A). However, if the droplet impacts on an asymmetric half-arc pattern with a curved opening facing right, it would rebound vertically upward and deflect to the right, and vice versa (Fig. S3B,C). Furthermore, when the wettability pattern has rotational symmetry like the pinwheel shape, the droplet would exhibit gyral rebounding. For example, when the droplet impacts the surface forming a circular film and reaching maximum spreading, it would rotate and rebound along with the hydrophilic patterns in a four-lobed pinwheel morphology due to the heterogeneously surface (Fig. S3D).

## Conclusion

Droplet-based transport driven by surface tension has been explored as an automated pumping source for several biomedical applications. This paper presented a fast and simple superhydrophobic modify and patterning approach to fabricate various open-surface platforms to manipulate droplets to achieve transport, mixing, concentration, and rebounding control. In our approach, several commercial reagents were utilized to create a superhydrophobic layer, and laser cutters are used to scan these superhydrophobic surfaces to generate gradient hydrophilic micro-patterns. Implementing back-and-forth vibrations on the predetermined parallel patterns, droplets can be transported and mixed successfully. Colorimetry of horseradish peroxidase (HRP) mixing with substrates also reduced the reaction time by more than 5 times with the help of superhydrophobic patterned chips. Besides, patterned superhydrophobic chips can significantly improve the sensitivity of colorimetric glucose-sensing by over 10 times. We expect this platform to achieve rapid surface modification and broaden to multiple kinds of bio-applications.

## Materials and methods

### Fabrication of the superhydrophobic film

**Figure 6:**
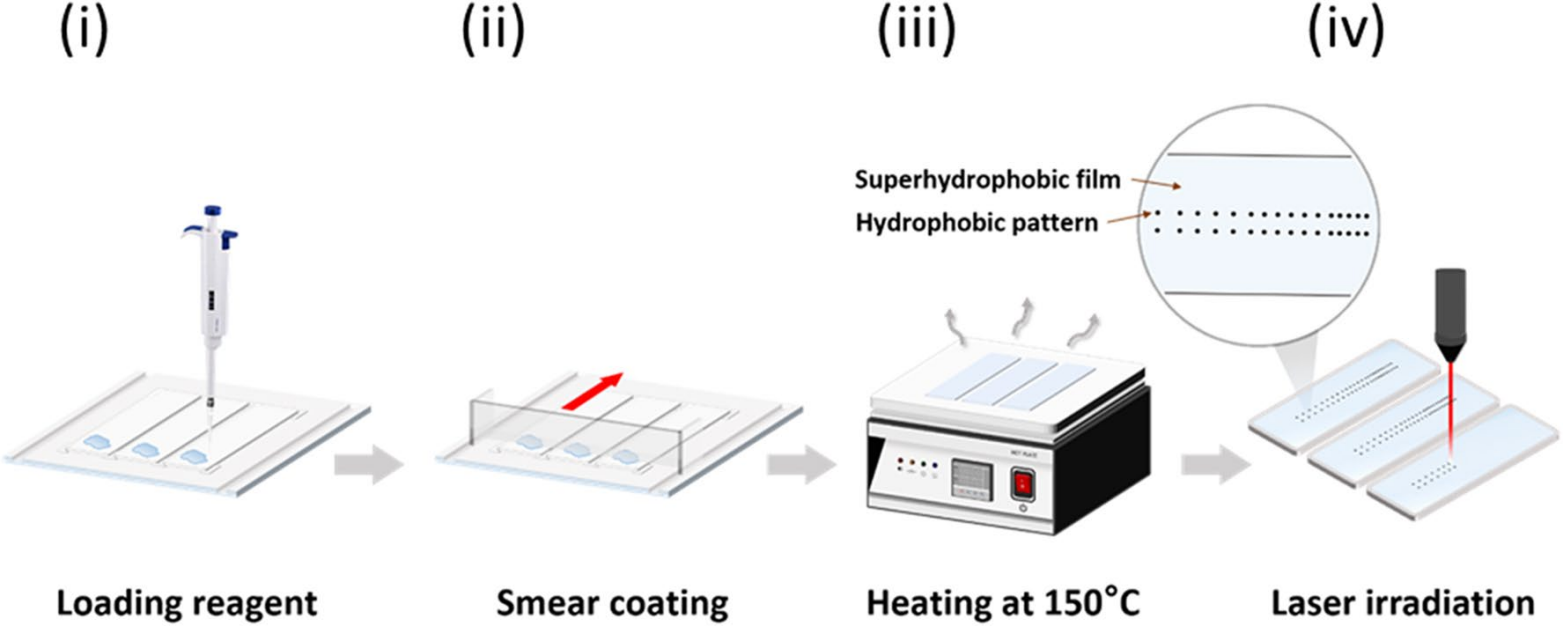
Fabrication of superhydrophobic film with gradient wettability patterns. (i) Loading the superhydrophobic reagent on the glass. (ii) Using the smear coating method to uniformly coat the surface. (iii) Heating the glass at 150 °C for 1 h. (iv) Lasering hydrophilic patterns on superhydrophobic surface to create wettability substrates.

Several commercial reagents were tested in our approach, and the Glaco reagent was selected to create a superhydrophobic layer (Table S1). The glass slide is coated with the superhydrophobic film using a commercial coating agent (Glaco Mirror Coat “Zero”, Soft 99 Co., Japan), which is a suspension of silica nanoparticles hydrophobically modified with alkyl functions in isopropyl alcohol. The coating procedure is a simple three-step process, as shown in Fig. 6. First, we load the Glaco solution on the glass, then use a smear device fabricated by our research team to get a uniform coating. Finally, the coating is stabilized by heating the glass at 150 °C for 1 h. After coating, we get a superhydrophobic surface and with a static contact angle of approximately 151°. Related steps and optimization are described in Figure S4.

### Wettability micropattern

After coating the superhydrophobic film on the glass, we create the superwettable micropattern by a 60 W CO_2_ laser-cutting machine (Taiwan 3 Axle Technology Co., Ltd./DC5030B). The laser is used to fabricate the hydrophilic gradient microstructures to change the degree of hydrophobicity. All the hydrophilic micropatterns consist of point cascades within rectangular areas. We get four different regions of a pattern with gradient surface wettability by adjusting the intervals of point cascades (0.5, 0.75, 1, and 1.25 mm). Here, we use the hydrophilic ratio to define the capability of the hydrophilic micropattern to grab the droplet on the superhydrophobic surface. As the distance of point intervals decreases, the hydrophilic ratio increases. For example, the point interval of 0.5 mm in this point cascade represents the more hydrophilic edge within a pattern, and the point interval of 1.25 mm represents the more hydrophobic edge. We define the surface wettabilities by contact angle measurement of 20 μL water droplets with ImageJ software. Other laser operating parameters, including scanning time (0.01 s), traveling speed (100 mm/s), and resolution (100 ppi) of the point laser, are kept constant throughout the experiments. Each area is 1 mm × 16 mm unless otherwise noted.

### Working mechanism

The motorized actuation system is a platform creating back-and-forth vibrations to manipulate the movement of the droplets on the superhydrophobic surface with hydrophilic micropatterns. Once the vibration is applied, droplets are controlled by the inertial force and the drag force. Due to the gradient of the surface wettability, the droplet on the micropattern can move spontaneously from the hydrophobic edge to the hydrophilic edge.

### Enzyme colorimetric activity

TMB (tetramethyl benzidine, Sigma Aldrich) as a substrate for HRP (horseradish peroxidase, Sigma Aldrich) was purchased for the enzyme colorimetric activity. HRP was dissolved in phosphate buffer (50 mM, pH 6.5) to get the HRP solution (2 mg/mL). Next, 20% glycerol was mixed with HRP solution to get the HRP/glycerol solution (0.01 mg/mL). After the H_2_O_2_ and TMB solution were added to HRP/glycerol solution for coloring, oxidation of TMB by HRP/H_2_O_2_ generates a blue-colored complex product, which turns yellow after the addition of sulfuric acid to the reaction media. Finally, results of enzyme colorimetry were photographed using a smartphone camera (iPhone 7, Apple Inc., USA) then processed with ImageJ for data analysis.

### Glucose colorimetric activity

Glucose samples at concentrations of clinically relevant ranges (2.5 to 50 mM) were prepared for glucose colorimetric activity. A mixture of enzyme solution contained 100 units of horseradish peroxide (HRP) and 500 units of gluxcose oxidase (GOx) was dissolved in 39.2 mL phosphate buffer of deionized water. This enzyme solution was stored in a dark environment at − 20 °C. Before the experiment, diluted 10:1 with the enzyme solution of HRP/GOx in a chromogen (potassium iodide, 0.3 M) and kept at 4 °C. The HRP, with hydrogen peroxide, oxidizes potassium iodide to elemental iodine (yellow to brown color). The color intensity was digitally scanned with a photo scanner (iPhone 7, Apple Inc., USA). The resulting image was converted to a gray scale and analyzed for intensity using Image-J histogram software (v 1.8.0).

## Supplementary Information

Supplementary Information.
