# Supplementary material for: Rapid construct superhydrophobic microcracks on the open-surface platform for droplet manipulations
Source: Sci Rep. 2021 Jul 21;11:14915. doi: 10.1038/s41598-021-94484-y (PMC8295315; doi:10.1038/s41598-021-94484-y)
Supplement: Supplementary file 1 — Supplementary Information. [file 41598_2021_94484_MOESM1_ESM.pdf]

Supporting information

Rapid construct superhydrophobic microcracks on the open-surface platform for droplet manipulations

Wan-Hsuan Lin[1], Chien-Wei Chen[2,3], Sheng-Hang Wang[2], Bor-Ran Li[1, 2, 4] *

1. Institute of Biomedical Engineering, College of Electrical and Computer Engineering, National Yang Ming Chiao Tung University, Hsinchu, Taiwan.
2. Department of Electrical and Computer Engineering, College of Electrical and Computer Engineering, National Yang Ming Chiao Tung University, Hsinchu, Taiwan.
3. Taiwan Instrument Research Institute, National Applied Research Laboratories, Hsinchu, Taiwan.
4. Center for Emergent Functional Matter Science, National Yang Ming Chiao Tung University, Hsinchu, Taiwan

* To whom correspondence should be addressed.

Dr. Bor-Ran Li

Address: Institute of Biomedical Engineering, National Yang Ming Chiao Tung University, 1001 Ta-Hseh Rd. Hsinchu, Taiwan

Tel No: 886-3-5712121 ext. 54051

Fax No: 886-3-5165993

E-mail: liborran@nycu.edu.tw

Table. S1 *Compare the contact angle of water on the glass slide coating with various reagents.*

**Reagents**	**Glass**	**Hoda**	**Crep**	**NeverWet**	**Glaco**
**10 µl**	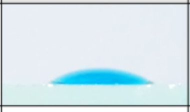	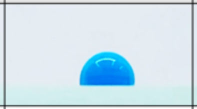	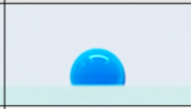	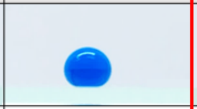	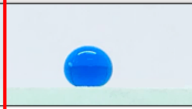
Contact angle	22 ±5°	97 ±1°	106 ±0.3°	140 ±2°	150 ±0.5°
Degree of hydrophobicity	-	+	+	++	++

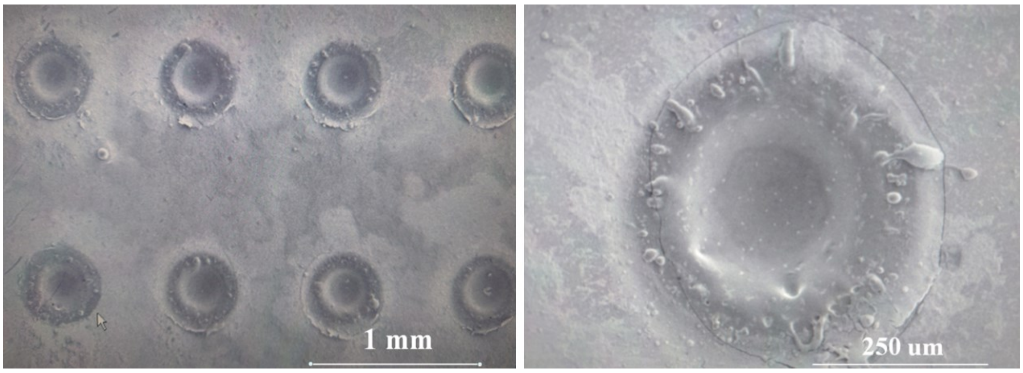

Fig. S1 *SEM images of the arrangement of points created by laser cutter.*

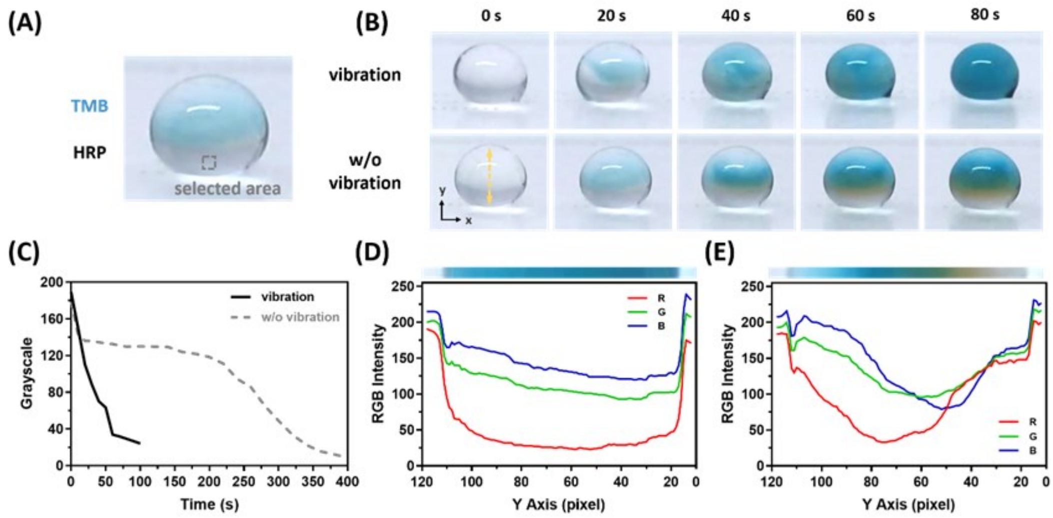

Fig. S2 *(A) The droplet mixing with HRP determination. (B) Time-lapsed images of the droplet mixing process. (C) Calculating the selected area to present the droplet mixing efficiency. The distributions of RGB intensity along the yellow dashed line (Fig. 5B) indicate the color of the mixture droplet changing under 80 s with (D) and without (E) vibration.*

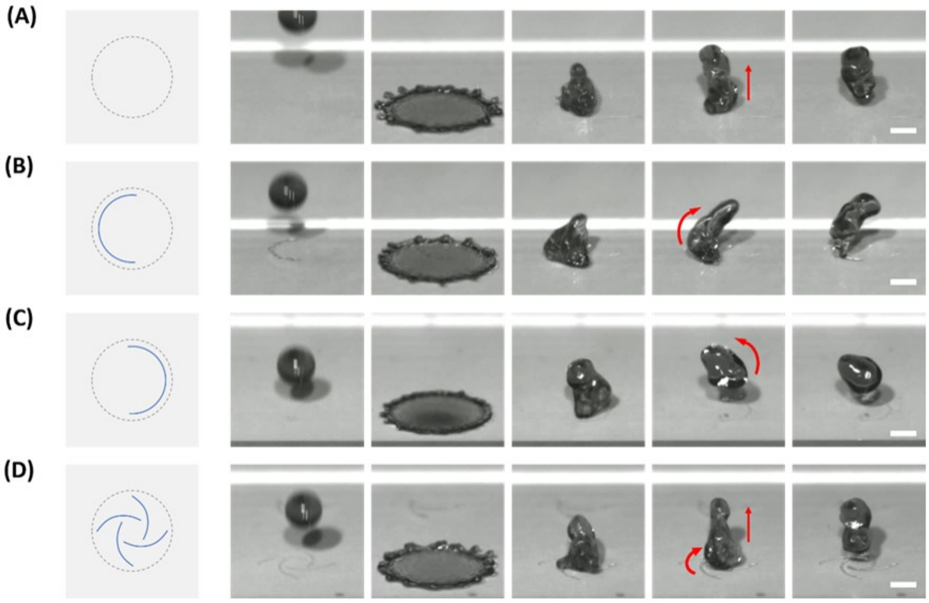

Fig. S3 *(A) Droplet impacted the superhydrophobic surface without any wettability patterns (rebounding vertically upward). (B) Droplet impacted an asymmetric half-arc pattern with the curved opening facing the right (rebounding vertically upward and deflecting to the right). (C) Droplet impacted an asymmetric half-arc shape pattern that curved opening facing left, it rebounds vertically upward and deflects to the left side. (D) Droplet impacted on the pinwheel-like pattern (rotating and rebounding along the hydrophilic patterns). The scale bar is 2 mm.*

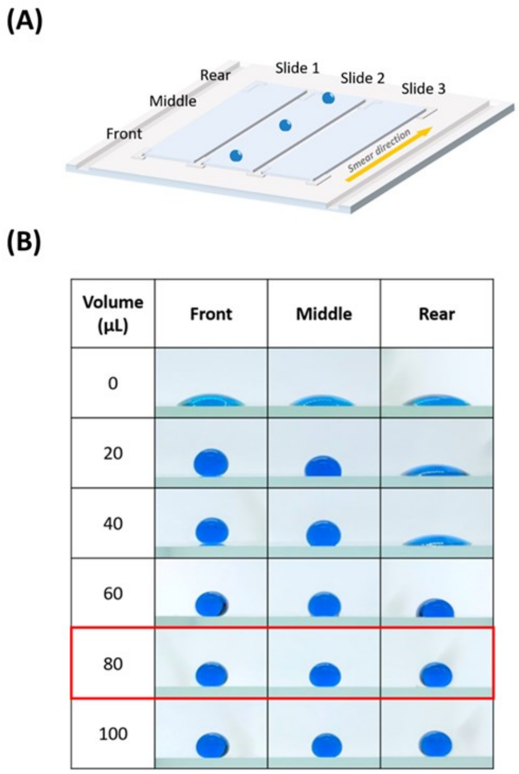

Fig. S4 *(A) Illustration of smear coating method to uniformly coat the surface. (B) Summary of the effect of reagent volume and surface homogeneity. The red square indicates the optimized conditions.*
